# The Use of Computational Methods for the Development of Molecularly Imprinted Polymers

**DOI:** 10.3390/polym13172841

**Published:** 2021-08-24

**Authors:** Ian A. Nicholls, Kerstin Golker, Gustaf D. Olsson, Subramanian Suriyanarayanan, Jesper G. Wiklander

**Affiliations:** Bioorganic & Biophysical Chemistry Laboratory, Linnaeus University Centre for Biomaterials Chemistry, Department of Chemistry & Biomedical Sciences, Linnaeus University, SE-391 82 Kalmar, Sweden; kerstin.golker@lnu.se (K.G.); gustaf.olsson@lnu.se (G.D.O.); esusu@lnu.se (S.S.); jesper.wiklander@lnu.se (J.G.W.)

**Keywords:** chemometrics, computational chemistry, density functional theory, molecular dynamics, molecular imprinting, molecularly imprinted polymer, multivariate analysis

## Abstract

Recent years have witnessed a dramatic increase in the use of theoretical and computational approaches in the study and development of molecular imprinting systems. These tools are being used to either improve understanding of the mechanisms underlying the function of molecular imprinting systems or for the design of new systems. Here, we present an overview of the literature describing the application of theoretical and computational techniques to the different stages of the molecular imprinting process (pre-polymerization mixture, polymerization process and ligand–molecularly imprinted polymer rebinding), along with an analysis of trends within and the current status of this aspect of the molecular imprinting field.

## 1. Introduction

Molecular imprinting has been defined as: “The construction of ligand selective recognition sites in synthetic polymers where a template (atom, ion, molecule, complex, or a molecular, ionic or macromolecular assembly, including micro-organisms) is employed in order to facilitate recognition site formation during the covalent assembly of the bulk phase by a polymerization or polycondensation process, with subsequent removal of some or all of the template being necessary for recognition to occur in the spaces vacated by the templating species” [1].

**Figure 1:**
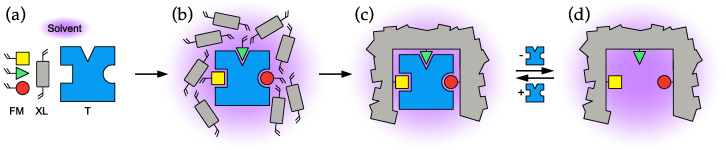
Schematic description of the different stages in the molecular imprinting process. (**a**) The main polymer components: template (T), functional monomers (FM) and cross-linking monomer (XL). (**b**) Pre-polymerization mixture, (**c**) after polymerization, (**d**) after template removal.

The most central feature of the molecular imprinting concept [1,2,3,4,5] is the interaction between template and monomers in the pre-polymerization mixture (Figure 1b) and their effect on the structure and recognition properties of the resulting molecularly imprinted polymer (MIP), as shown in Figure 1.

Consequently, the molecular and physical characteristics of recognition sites in MIPs result directly from the various interactions possible in the pre-polymerization mixture, e.g., template–monomer, monomer–monomer, solvent–template/monomer, etc. An appreciation of the physical rules governing the formation of these complexes is therefore crucial for understanding the complexity of the imprinting process. If we are to achieve true rational design of molecularly imprinted systems for producing materials with predetermined recognition properties, suitable tools that can provide insight into the molecular recognition processes are needed.

Although classical thermodynamic models [6] in theory can describe the molecular events governing the synthesis and polymer–ligand recognition properties of imprinted materials, modern computational methods can be used to model the pre-polymerization mixture in much greater detail and even to characterize polymer–ligand interactions [7,8,9]. In this review, we first provide a brief background to the thermodynamic factors and theories that have been presented as a basis for explaining the recognition properties of MIPs, before reviewing the literature describing the use of computational methods for the study of the various stages of the molecular imprinting processes.

### 1.1. A Thermodynamic Treatment of the Molecular Imprinting Process

The physical factors underlying molecular interaction have attracted the interest of researchers for several decades. Jenck’s paradigms [10,11], the factorization of energetic contributions to molecular recognition and the intrinsic binding energy concept, are of particular note, and were employed by a number of groups. Semi-quantitative approaches, so-called back of the envelope calculations [12], were independently formulated by Andrews [12] and Williams [13,14,15], aspiring to define the physical basis for binding events. Nonetheless, as reflected in several studies [6,16,17], the thermodynamic factors controlling molecular interactions in imprinted systems are best described by Williams’ more comprehensive treatment [13,18] (Equation (1)):

Δ*G*_bind_ = Δ*G*_t+r_ + Δ*G*_r_ + Δ*G*_h_ + Δ*G*_vib_ + ∑Δ*G*_p_ + Δ*G*_conf_ + Δ*G*_vdW_ (1)

where the Gibbs free energy change for complex formation (Δ*G*_bind_) is the combined energy changes associated with the loss of translational and rotational freedom (Δ*G*_t+r_), restriction of rotors upon complexation (Δ*G*_r_), hydrophobic interactions (Δ*G*_h_), residual soft vibrational modes (Δ*G*_vib_), the sum of interacting polar group contributions (∑Δ*G*_p_), adverse conformational changes (Δ*G*_conf_) and unfavorable van der Waals interactions (Δ*G*_vdW_).

The recognition properties of MIPs result from pre-polymerization complexation between template and functional monomer, an equilibrium process governed by the free energy of binding, Δ*G*_bind_. The position of this equilibrium dictates the number and heterogeneity of the resulting binding sites. Stronger and more regular template-functional monomer complexes are thus expected to lead to a larger number of sites with higher fidelity. The degree of template complexation by a functional monomer and the degree of heterogeneity are determined by the chemical nature of the pre-polymerization mixture and the polymerization conditions (temperature and pressure).

An investigation of changes in NMR chemical shifts and line broadening with increasing functional monomer concentration offered the first direct verification of the formation of non-covalent template-functional monomer complexes [19]. This study also indicated possible template self-association and formation of higher-order complexes, a hypothesis that was more recently supported by computational studies based on molecular dynamics (MD) [20]. Spectroscopic methods have since been used in many studies aiming to shed light on the multitude of pre-polymerization equilibria involving template-functional monomer complexation [21,22,23,24,25,26,27], self-association [27] and interactions with cross-linking monomers [28]. In such studies, it is often seen that using higher ratios of functional monomer to template, in order to increase complex formation, leads to MIPs with a higher degree of non-specific binding.

Complex formation between a template and a functional monomer carries with it an entropic penalty, Δ*G*_t+r_, associated with the loss of translational and rotational freedom. Higher-order complexes, expected to produce higher-fidelity binding sites, thus have a larger energy barrier. It follows that using a functional monomer capable of multiple simultaneous interactions should produce increased concentrations of complexed template, compared to an increased concentration of a single-point monomer. Although multi-dentate monomers are not as easily available and often require synthesis, examples of their use in MIPs have been reported [29,30,31,32].

Similar to Δ*G*_t+r_, the Δ*G*_r_ term is the penalty for restricted internal bond rotation upon complexation. Thus, interactions with rigid templates are entropically favored and the resultant MIPs tend to exhibit higher selectivity than those prepared with less rigid structures. In addition, a rigid structure can adopt fewer solution conformations, which leads to a narrower site distribution. Consequently, high MIP–ligand affinities have been observed for rigid templates, such as the alkaloids morphine [33] and yohimbine [34].

MIPs have traditionally been prepared in non-polar organic media, thus relying on polar interactions to drive the equilibrium towards complexation, as reflected in the ∑Δ*G*_p_ term. Examples have been reported where selectivity was enhanced by using more strongly interacting monomers [35,36,37,38], or by using crown ethers to solubilize zwitterionic template–monomer complexes at low polarity [39]. In addition, several studies have reported the use of metal ions to provide multiple coordination points between template and monomers, enabling the use of more polar solvents such as methanol or DMSO [40,41,42,43,44].

When the analyte of interest is water-soluble or otherwise incompatible with the commonly used non-polar organic solvents, other methodologies are required. For many important classes of analytes, e.g., peptides, proteins, oligonucleotides and sugars, the use of water as the porogen (solvent of polymerization) places a strong influence on the Δ*G*_h_ term of Equation (1). This enables functional monomers with hydrophobic moieties to facilitate template complexation through the hydrophobic effect. Some interesting examples have been reported where polymerizable cyclodextrins were used as monomers [45,46,47,48,49]. In addition, metal ion chelation can be used as an alternative or complement.

In summary, for non-covalently imprinted polymers, the selectivity and affinity of the MIP is controlled by the various equilibria present in the pre-polymerization mixture. The positions of these equilibria are, in turn, governed by Δ*G*_bind_, as defined by the different thermodynamic terms in Equation (1). The magnitudes of the individual terms are determined by the chemical nature of all components in the mixture as well as the physical conditions during polymerization. Thus, the conceptually simple process of molecular imprinting is based on a complex series of highly interdependent equilibria, inevitably leading to polymers with heterogenous distributions of recognition sites. The same thermodynamic principles, of course, also apply to interactions between the polymer and its analyte/target in the intended application, further complicating the link from pre-polymerization conditions, over polymerization and work-up, to final use. Accordingly, there is a need for methodologies to study and understand the complexity of pre-polymerization events, to correlate these events with MIP performance and to optimize all stages of MIP synthesis.

### 1.2. Theoretical and Computational Strategies for MIP Development

**Figure 2:**
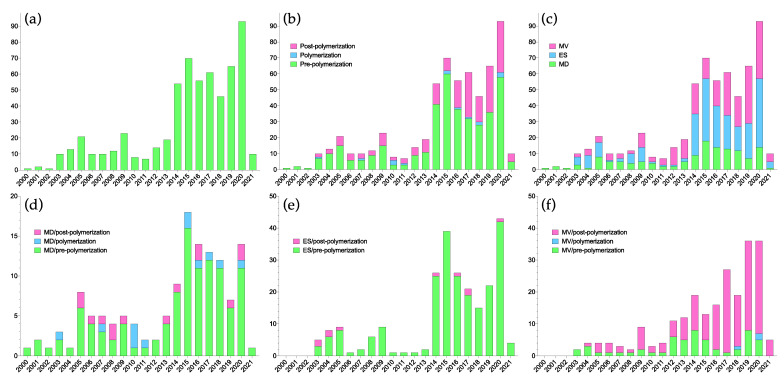
Number of papers published where computational methods have been applied to some aspect of molecular imprinting. (**a**) Total number of papers, (**b**) number of papers according to stage, (**c**) number of papers according to method: multivariate (MV), electronic structure (ES) or MD, (**d**) MD-based papers according to stage, (**e**) ES-based papers according to stage and (**f**) MV-based papers according to stage. Note that a number of papers fall into more than one category.

Driven by the rapid development of molecular imprinting and its applications, several tools for in silico studies of the above-mentioned stages of MIP design and synthesis have been adopted. These tools can offer atomistic insights on aspects ranging from events in the pre-polymerization mixture to polymer–ligand interactions and even polymer morphology. Since the first applications of computational strategies to study aspects of molecular imprinting in the beginning of the 21st century, the field has grown steadily, and particularly over the last decade (Figure 2). This development has been supported through the necessary iterative interplay between these studies and experimental validation.

In the past, knowledge of the molecular events underlying MIP behavior has been extracted from empirical studies of polymer–ligand interactions. Thermodynamic models [6,16,17,50,51], as discussed above, were applied in attempts to explain and understand both pre-polymerization events as well as polymer recognition characteristics. More recently, probability-based stochastic simulations of pre-polymerization monomer–template equilibria [52] contributed to this area, an example of which was the use of a stochastic algorithm [53] to simulate pre-polymerization solution heterogeneity, placing monomer-template units in a lattice matrix. Importantly, the simulated affinity distributions closely matched those measured experimentally in MIPs. Additionally, mathematical models describing pre-polymerization template–monomer complexation and subsequent template rebinding have been developed [54].

The major reason behind the recent increase in the use of computational strategies in MIP technology is likely related to increased affordability of computational power and access to appropriate software [55,56,57,58]. This has enabled the application of multivariate analyses, electronic structure calculations and full-system all-atom MD simulations to all aspects of MIP design, synthesis and evaluation. After a brief introduction of the different computational tools, focusing on methods for electronic structure calculations, MD simulations and statistics-based multivariate analyses, we review the current status of their application to the different stages of molecular imprinting.

#### 1.2.1. Electronic Structure Calculations

The use of computational methods based on electronic structure calculations, e.g., ab initio, semi-empirical and density functional theory (DFT) strategies, for the design and evaluation of MIPs is increasing. This class of computational methods, collectively termed quantum chemistry, aims to solve the electronic Schrödinger equation based on the atomic coordinates and number of electrons of the system studied. This is impossible for systems with more than a few electrons, and therefore approximations are necessary. Ab initio methods approximate the electronic wavefunction, whereas semi-empirical and DFT methods instead approximate the Hamiltonian operator. The accuracy, and computational demand, increases from semi-empirical methods (considering only valence electrons and with some parameters derived from experiment) to DFT (calculates electron densities), and finally ab initio methods. Different strategies, basis sets and parameters are chosen to provide an acceptable approximation of the system studied within a reasonable timeframe. Typically, comparing quantum chemical calculations for isolated molecules and molecular complexes can provide information regarding interaction strength and type, and consequently, these methods are very often used for evaluation of different template–monomer combinations.

#### 1.2.2. Molecular Dynamics

Although the development of modern MD methodology was closely intertwined with that of Monte Carlo simulations [59,60,61,62,63], it is generally considered as introduced by Alder and Wainright in a seminal study involving hard sphere simulations of gaseous argon in 1957 [64]. Other key developments include the transition to MD simulations of liquid argon by Rahman in 1964 [65], as well as liquid water simulations in 1971 by Rahman and Stillinger [66]. In MD simulations, the forces acting on and between interacting atoms and molecules are described by a set of equations and parameters, referred to as a force field [67]. Solving Newtons equations of motion allows for simulation of the motions, or dynamics, of the system. MD simulations have been applied to an increasing number of research areas, driving the development of both software and force fields. Examples include studies of surfaces [68,69], solvents [70], biomolecular interactions [71,72,73], DNA conformation [74,75], protein folding [76], phospholipid bilayers [77,78] and membrane transport of drugs [79]. Some of the more popular and commonly used force fields are AMBER [80,81,82,83], GAFF [84], CHARMM [85], OPLS [86] and GROMOS [87,88].

**Figure 3:**
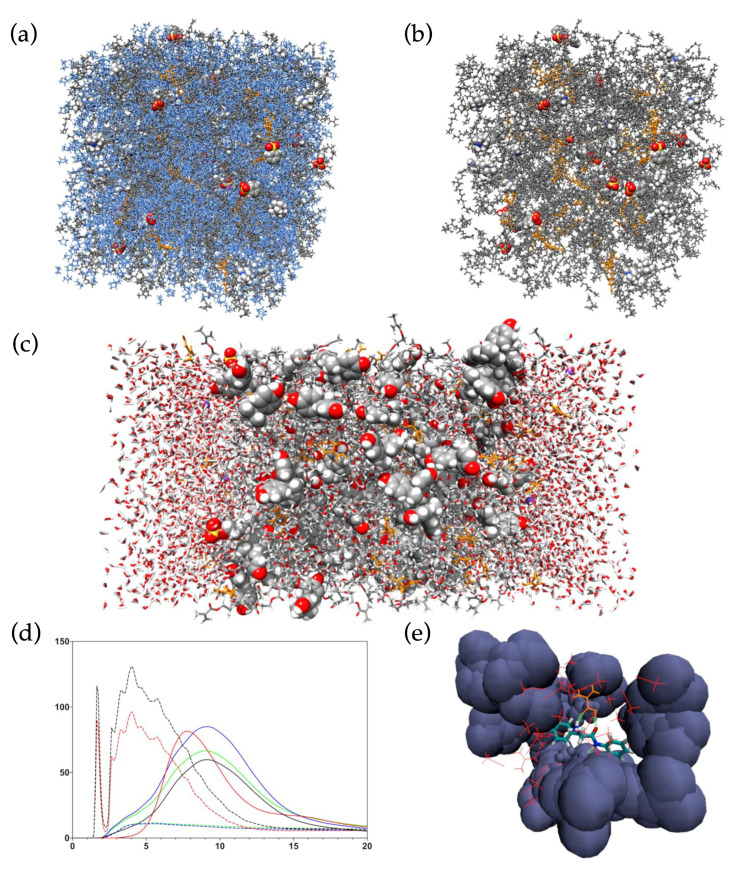
Examples of MD simulation systems and MD data-derived analysis methods. (**a**) Full-system all-atom MD simulation of urea-based MIP anion receptors [89]. (**b**) The same system though excluding solvent (tetrahydrofuran) from visualization. (**c**) Two-phase water bisphenol A MIP emulsion polymerization simulation [90], where polymer components are flanked by aqueous phase with dissolved counter ions. (**d**) Radial distribution function analyses for component interactions [89]. (**e**) Grid density analysis plot, where local densities of interacting species are visualized [20].

In comparison to electronic structure methods, MD simulations require less computational resources when treating systems of comparable size. This enables studies of much larger multimolecular systems, including MIP pre-polymerization mixtures containing thousands of molecules, with reasonable demands on both hardware resources and time. However, since electrons are not explicitly considered, MD simulations are unable to account for processes involving the movement of electrons, such as bond breaking or formation. Nevertheless, important information can be attained regarding the multitude of non-covalent interactions taking place in pre-polymerization mixtures as well as in MIP binding site models (Figure 3).

#### 1.2.3. Multivariate Analysis

Molecular imprinting and its applications, with nearly infinite combinations of pre-polymerization components, polymerization conditions, polymer workup, evaluation parameters and analytic responses, lends itself well to multivariate analysis [91,92,93]. This entails a different type of modeling than discussed above regarding molecular energies and interactions. Instead, the goal here is to produce mathematical models able to simultaneously correlate multiple experimental variables with one or more properties of a MIP and/or its application. The resultant models can then be used to optimize, e.g., the polymer recipe or analytical parameters, or to find patterns and correlations hidden in large datasets.

**Figure 4:**
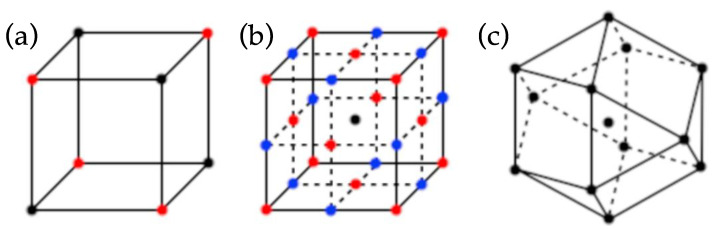
Schematic illustrations of examples of experimental design concepts/methods used in the statistical evaluation of MIPs. Examples of controllable variables include, amounts of functional monomer, cross-linking monomer and porogen. (**a**) Black points represent experimental runs in a three-factor fractional factorial design, while the combined red and black points represent a three-factor full factorial design. (**b**) Red and central black points depict a three-factor central composite design, while blue and central black points represent a Box-Behnken design. (**c**) A three-factor Doehlert design.

Application of multivariate methods usually begins with determining which parameters to study and then choosing an experimental design that allows for simultaneous evaluation of these parameters. Often, a pilot or training set of experiments is performed to determine which of the variables have the largest effect on the outcome, followed by a more focused, second experimental design in order to produce models for prediction or optimization. Common experimental designs include full or fractional factorial, Box-Behnken, Placket-Burman, Doehlert and central composite designs (Figure 4).

The experimental data can be calibrated, or fitted, to mathematical models using a number of methods. In MIP studies, the most common are principal component analysis (PCA), partial least squares regression (PLSR), multiple linear regression (MLR) and artificial neural networks (ANN).

## 2. The Pre-Polymerization Stage

As seen in Figure 2b, the majority of papers employing computational treatments have focused on the pre-polymerization stage, predominantly using electronic structure methods or MD simulations.

### 2.1. Electronic Structure Calculations

Demands on hardware resources and simulation time both increase rapidly with the size of the system under investigation. Accordingly, the most common use of electronic structure-determining methods in MIP studies for characterization of template–monomer complexes, as discussed above, is to find the most suitable functional monomer and often also the optimal stoichiometry. Of the electronic structure-determining methods, semi-empirical strategies are less demanding on computational resources, and the two most commonly used semi-empirical methods for MIP development are AM1 [94,95,96,97,98,99,100,101,102,103,104,105,106] and PM3 [107,108,109,110,111,112,113,114,115,116,117,118,119,120,121,122,123,124,125,126,127], while other examples are less common [128,129,130].

The more computationally demanding ab initio and DFT methods provide higher accuracy. Investigations involving template–monomer complex studies have employed different methods, basis sets and levels of theories on several occasions. The majority of these studies employed DFT methods [124,131,132,133,134,135,136,137,138,139,140,141,142,143,144,145,146,147,148,149,150,151,152,153,154,155,156,157,158,159,160,161,162,163,164,165,166,167,168,169,170,171,172,173,174,175,176,177,178,179,180,181,182,183,184,185,186,187,188,189,190,191,192,193,194,195,196,197,198,199,200,201,202,203,204,205,206,207,208,209,210,211,212,213,214,215,216,217,218,219,220,221,222,223,224,225,226,227,228,229,230,231,232,233,234,235,236,237,238,239,240,241,242,243,244,245,246,247,248,249,250,251,252,253,254,255,256,257,258,259,260,261,262,263,264,265,266,267,268,269,270,271,272,273,274,275,276,277,278,279,280,281,282,283,284,285,286,287,288,289,290,291,292,293,294,295,296,297], whereas ab initio-based calculations have been used in fewer instances [298,299,300,301,302,303,304,305,306,307,308,309,310,311,312,313,314,315,316,317,318,319,320,321,322,323]. The majority of these studies focus on the interaction between a single functional monomer and a single template. In one study, semi-empirical, DFT and ab initio-based calculations were compared for characterization of monomer–template interactions [324].

The growing accessibility of computational power has been accompanied by an increasing frequency of reports in the literature including quantum chemical calculations in the design and characterization of MIPs. Due to relatively high resource requirements associated with these calculations, most studies have focused primarily on subsets of pre-polymerization mixtures, restricted sets of interactions or isolated non-solvated molecular complexes in vacuo. With seemingly ever-increasing availability of computational power and emergence of novel mixed approaches combining electronic structure and MD simulations, increased use of electronic structure methods in the design and study of molecular imprinting systems is expected.

### 2.2. Molecular Dynamics

The nature of MD simulations makes them highly suited for studies of liquid systems, and with the assumption that MIP recognition properties originate from pre-polymerization interactions [4,51,325,326,327], MD simulations have primarily found applications in studies of this stage of MIP production. From the resulting data, or molecular trajectories, information regarding the types and strengths of all pre-polymerization interactions can be extracted and correlated with MIP recognition performance. Since the computational cost is significantly lower for force field methods than for quantum chemical calculations, MD simulations can be applied to very large systems with solvent molecules explicitly included.

In a method introduced by Piletsky et al., 20 functional monomers were initially assessed for their interaction energy with the template ephedrine in both charged and neutral states [328]. Selected monomers were then used for polymer synthesis, but also subjected to further MD simulations together with template, cross-linker and solvent, where the observed interactions could be correlated with experimental binding data. This approach has since been adapted several times in the literature [329,330,331,332,333,334,335,336,337,338,339,340,341,342,343,344,345,346,347,348,349,350,351,352,353]. In a number of reports, similar approaches have been employed to evaluate and/or characterize monomer–template interactions using MD and docking simulations as well as variations and/or combinations thereof [102,354,355,356,357,358,359,360,361,362,363,364,365,366,367,368,369,370,371,372,373,374,375,376,377,378,379,380,381,382,383,384,385,386,387,388,389,390,391].

Despite the dramatic development of computer hardware and software, multimolecular simulations involving multiple copies of monomers, template and explicit solvent are still not feasible for electronic structure methods alone. However, several examples report the combined use of quantum chemical calculations and MD simulations to study different aspects of pre-polymerization mixtures [128,143,215,216,392,393,394].

The growing number of MD studies of systems containing all MIP components and with experimental stoichiometries have highlighted the importance of these more comprehensive treatments of pre-polymerization mixtures for delineating underlying mechanisms [20,28,89,90,201,395,396,397,398,399,400,401,402,403,404,405,406,407,408,409,410,411,412,413].

MD-based investigations of other aspects of the pre-polymerization stage include studies of the structural stability of protein epitopes for template screening [414,415], mapping potential monomer interaction sites of a protein target, followed by docking of acrylamide-derived monomers and post-docking interaction energy calculations [416], studies of template interactions with Dengue virus as a support matrix to create larger binding sites [417], a series of reports attempting to correlate structural and physical properties of dummy templates and ligands with rebinding properties [418,419,420,421,422,423,424,425,426] and coarse-grained simulations studying the effect of composition on material properties and template interaction [427]. Additionally, large-scale MD simulations were performed in an attempt to mimic chromatography in a virtual capillary [428].

### 2.3. Multivariate Analysis

Traditionally, analysis and optimization of MIPs have been univariate in nature. This involves evaluation and optimization of one parameter with the results carried forward for optimization of the next, and so forth. This may not always be ideal as identified optima may turn out to be local or false [91]. The inherent flexibility of MIP synthesis and the interdependence of the variables makes this stage a good candidate for multivariate optimization. Consequently, a number of studies have been published applying different multivariate methods and experimental designs in order to optimize polymer composition and/or synthesis methods [210,211,212,213,305,429,430,431,432,433,434,435,436,437,438,439,440,441,442,443,444,445,446,447,448,449,450,451,452,453,454,455,456,457,458,459,460,461,462,463,464,465,466,467,468,469,470,471,472,473,474,475,476,477,478].

## 3. The Polymerization Stage

The polymerization reaction is the least studied aspect of molecular imprinting in general as well as in the context of computational treatment (Figure 2b). The imprinting literature is abundant with experimental correlations between pre-polymerization mixture composition and MIP recognition properties, providing support for the underlying assumption that template-functional monomer complexes are preserved in the polymer matrix. However, little direct evidence exists regarding the fate of these complexes once polymerization has been initiated, though NMR studies indicate that they are maintained during polymerization [23]. Nevertheless, there are examples of studies of the polymerization stage by means of computational methods, almost exclusively using molecular dynamics (Figure 2d). The development of reactive force fields [479,480] and other solutions enabling bond formation and breaking in force field-based simulations should further help in filling this knowledge gap.

### 3.1. Electronic Structure Calculations

Although this class of computational methods can accurately describe the movement of electrons and the breaking and formation of chemical bonds, the number of molecules required for a meaningful representation of the polymerization process of a MIP would lead to unreasonable computational demands. Hopefully, the technical development will eventually allow such calculations, yet to the best of our knowledge, no examples have been published.

### 3.2. Molecular Dynamics

A few attempts have been made to apply MD to the study of MIP polymerization. Yungerman and Srebnik used a coarse-grained Monte Carlo procedure to study the formation of binding site imperfections in MIPs [481]. Monomers were modeled as Lennard-Jones spheres and templates as rigid dumbbells made of two monomers. Consequently, the simulations only considered imprinting according to size and shape. Other similar studies have also been reported, combining Monte Carlo simulation of hard spheres with statistical mechanics [482,483,484,485,486,487] or mean field theory [488,489,490,491,492]. However, in order to replicate MIP recognition on a molecular level, it is necessary to perform atomistic simulations. Thus, Henthorn and Peppas reported all-atom MD-based simulations of the formation of glucose-imprinted polymers [493,494], where 160 template molecules, 160 functional monomers (2-hydroxyethyl methacrylate), 300 cross-linkers (ethylene glycol dimethacrylate, EGDMA), 800 water molecules and 20 initiator molecules were allowed to diffuse and relax using MD simulation, followed by a reaction step including initiation, propagation and termination. This was repeated until all radicals had been quenched. Ligand binding to the resultant polymer models was then compared with experimental data. Srebnik and co-workers combined a similar reaction scheme with lattice Monte Carlo simulations in a series of studies of protein-imprinted polymers [427,495,496,497,498,499]. Schauperl and Lewis attempted to simulate the polymerization reaction for xanthine MIPs [500]. Starting with one or more template molecules, monomers and cross-linkers were sequentially added to the system and allowed to form new bonds with the growing polymer chain. MD and energy minimization allowed for optimal host–guest interaction. The simulations were continued until a threshold density had been reached. The resultant polymer model was used to explain binding site heterogeneity. Efforts to simulate electropolymerized MIPs selective for 6-thioguanine were reported by Hyunh et al. [501]. A system with one template molecule, two functional monomers and six cross-linking monomers was subjected to MD simulation. Pre-determined “radical positions” in the monomers were allowed to form bonds if within a 3 Å distance until no additional bonds were formed. The equilibrated system was then replicated eight times, whereafter the MD simulation continued until saturation. No analysis of the resultant model was reported other than that its density was very similar to that of the polymers prepared by electropolymerization. Cowen and co-workers developed a similar algorithm for simulating the polymerization reaction during MD simulations of the pre-polymerization mixture [502,503]. Briefly, after equilibration of the system, new bonds were formed between “reactive” atoms within a suitable distance followed by another round of energy minimization. The process was repeated until no more reactions were possible.

### 3.3. Multivariate Analysis

The application of multivariate strategies in order to optimize polymerization conditions has been rare so far. Examples include studies of optimum polymerization temperature when comparing polymerization in bulk and surface molecular imprinting to study the role of insulin-imprinted magnetic nanoparticles [469], investigation of the influence of polymerization temperature and time on the diameter of 5-fluorouracil-imprinted MIP nanoparticles prepared via precipitation polymerization [504] and optimization of the number of cycles and scan rate in electropolymerization of ketorolac tromethamine MIPs on paper graphite electrodes [505]. It should be noted that in many of the reports discussed in Section 2.3 attempting to optimize polymer synthesis, polymerization parameters were initially included in the experimental designs. However, when it was found that variation of these parameters had no significant influence on the outcome, they were omitted from further optimization.

## 4. MIP Structure and Function

The bulk of the computational studies of MIP properties post-polymerization use multivariate analysis, though a handful of reports using quantum chemical calculations or MD simulations have also been presented (Figure 2d–f). This is not surprising considering the opportunities for optimization of experimental parameters at this stage, e.g., rebinding conditions.

### 4.1. Electronic Structure Calculations

Electronic structure methods have been used in a few instances to study aspects of MIP–template recognition. These include PM3 calculations of a binding site model for nicotinamide [110], AM1 calculations to explain recognition differences in different buffers [506], DFT studies to explain the selectivity of a phenylurea herbicide MIP [507], DFT studies to confirm the structure of a binding site in a catalytic silica MIP [508], DFT studies of the adsorption mechanism in a 5-fluorouracil MIP [294,509], DFT studies of poly-pyrrole MIP models interacting with glyphosate [510] or tryptophan [511], DFT and ab initio studies of binding site models in hydroxyzine and cetirizine MIPs [299] and ab initio studies of a binding site model for phenolic compounds [512].

### 4.2. Molecular Dynamics

Attempts at simulating aspects of the rebinding of a template or ligand to a MIP using force field-based methods have also been reported. In several studies, polymer models have been approximated by equilibrating templates with linear chains of functional monomers, followed by analysis of binding energies and other aspects of recognition [355,356,358,513,514,515] or docking [221,360]. Terracina et al. also used docking procedures to study selectivity in MIP models that had been optimized semi-empirically [516]. Herdes and Sarkisov created pyridine MIP models by first equilibrating systems containing pyridine, methacrylic acid, EGDMA and chloroform [517]. The template and solvent were removed, and the monomers’ positions were fixed. Monte Carlo simulations were then applied to investigate the adsorption of pyridine, benzene and toluene. Sobiech et al. constructed MIP binding site models through MD simulation of pre-formed template/functional monomer/cross-linking monomer clusters [386,518,519]. After equilibration, the template was removed, and the system was “polymerized” by replacing double bonds in the monomers with new single bonds. A similar strategy was used by Gajda et al. to mimic an aripiprazole binding site [231]. Finally, Curk et al. developed a computational approach to derive binding site models and for evaluating template rebinding. Their approach used a range of parameters, including number of template interaction points, concentrations of monomers and material properties in combination with grand canonical Monte Carlo simulations describing multiple interaction site templates [54].

### 4.3. Multivariate Analysis

The experimental conditions employed when evaluating or applying MIPs have a major influence on the performance of the polymer. Similar to the situation in the pre-polymerization mixture, the possible combinations of experimental parameters (e.g., analyte concentration, solvent, pH, temperature, flow rate, incubation time) are nearly endless, making this an area highly suited for multivariate optimization. Thus, different combinations of experimental designs and response surface modeling have been used for optimization of parameters when using MIPs in adsorption, separation or sensing applications [214,267,375,467,468,470,475,476,477,520,521,522,523,524,525,526,527,528,529,530,531,532,533,534,535,536,537,538,539,540,541,542,543,544,545,546,547,548,549,550,551,552,553,554,555,556,557,558,559,560,561,562,563,564,565,566,567,568,569,570,571,572,573,574,575,576,577,578,579,580,581,582,583,584,585,586,587,588,589,590,591,592,593,594,595,596,597,598,599,600,601,602,603,604,605,606,607,608,609,610,611,612,613,614,615,616,617,618,619,620,621,622,623,624,625,626,627,628,629,630,631,632,633,634]. In some studies, the optimized parameters have been improved further by using them as input for ANN models [475,621].

In other cases, multivariate methods have been used to reveal correlations hidden in the data obtained when evaluating MIPs. Different combinations of PCA and PLSR methods have been applied for interpretation of MIP binding data obtained from SERS (surface-enhanced Raman spectroscopy) [635,636,637,638,639,640,641,642,643,644], for pattern recognition in the responses from various MIP-sensor arrays [645,646,647,648,649,650,651,652,653,654,655,656,657,658,659,660], to correlate the shape of MIP-quartz crystal microbalance frequency curves to different analytes [106,661,662,663], for cyclic voltammetry measurements on binary mixtures [214,656,664,665] and for correlating the results with HPLC data [665] and to process fluorescence data [666]. PLSR and PCA have also been used to correlate bupivacaine–MIP binding with rebinding solvent properties [667,668] and with polymer morphology and pre-polymerization interactions from MD simulations [403]. Likewise, PCA has been used to examine the relation between specific analyte sorption and non-specific sorption of water in an iprodione-imprinted MIP for use in aqueous media [669].

A separate branch of multivariate analysis of chemical data is called chemometrics, in which large numbers of structure-derived properties, molecular descriptors, are generated for a set of molecules and then correlated with other properties of interest. Thus, a number of studies have been reported attempting to correlate molecular descriptors with MIP binding data using a range of multivariate tools. Rossetti et al. employed PLS models to correlate molecular descriptors with solid-phase extraction retention data for a series of biomarker pro-gastrin-releasing peptides in order to elucidate the recognition mechanism [670]. Liu et al. used MLR and PCA to couple structural and molecular parameters of a quercetin MIP to its adsorption selectivity [671]. Baggiani et al. employed PCA to correlate the chromatographic selectivity of a pentachlorophenol MIP for the template and 52 related phenols with 16 AM1-derived molecular descriptors [672]. The chromatography data from this study was later subjected to PLSR modeling using 25 descriptors, which improved the selectivity prediction capability of the model [673]. Nantasemat et al. also built ANN models for prediction of MIP selectivity using molecular descriptors for a set of templates, functional monomers and HPLC mobile phases compiled from the literature [674,675], and for bisphenol A MIPs [676]. Similar models, attempting to correlate analyte recognition with molecular descriptors, have also been reported for MIPs selective for penicillin G [677], erythromycin [678] and milk lactose [679].

## 5. Conclusions and Outlook

The significant growth in the number of literature reports describing computational studies of molecular imprinting systems has followed the development, availability and affordability of both hardware and software. In turn, this has enabled the use of these tools in both prognostic and diagnostic capacities in the development of molecularly imprinted materials. This development and the growing awareness of the value of the use of these tools is reinforced through validation using experimental studies. Accordingly, the combination of ready access to these computational tools and the value of the insights gained from their use should see further increases in the prevalence of their use in the molecular imprinting field.
